# The medical futility experience of nursing professionals in Greece

**DOI:** 10.1186/s12912-021-00785-y

**Published:** 2021-12-20

**Authors:** Polychronis Voultsos, Anna Tsompanian, Alexandra K. Tsaroucha

**Affiliations:** 1 Laboratory of Forensic Medicine & Toxicology (Medical Law and Ethics), School of Medicine, Faculty of Health Sciences, Aristotle University of Thessaloniki, University Campus, 54124 Thessaloniki, GR Greece; 2 Postgraduate Program on Bioethics, Democritus University of Thrace, School of Medicine, Dragana, 68100 Alexandroupolis, GR Greece; 3 Postgraduate Program on Bioethics, Laboratory of Bioethics, Laboratory of Experimental Surgery and Surgical Research, Democritus University of Thrace, School of Medicine, Dragana, 68100 Alexandroupolis, GR Greece

**Keywords:** Nurses/nursing professionals, Futile medical care/treatment, Good/bad death, Dignified/undignified death, Suffering

## Abstract

**Background:**

Providing futile medical care is an ever-timely ethical problem in clinical practice. While nursing personnel are very closely involved in providing direct care to patients nearing the end of life, their role in end-of-life decision-making remains unclear.

**Methods:**

This was a prospective qualitative study conducted with experienced nursing professionals from December 2020 through May 2021. Individual in-depth qualitative interviews were conducted with sixteen participants. We performed a thematic analysis of the data.

**Results:**

Importantly, many participants were *half-hearted in* their *attitude towards* accepting or defining futile medical care. Furthermore, interestingly, a list of well-described circumstances emerged, under which the dying process is most likely to be a “bad and undignified” process. These circumstances reflected situations revolving around a) pain and suffering, b) treating patients with respect, c) the appearance and image of the patient body, and d) the interaction between patients and their relatives. Fear of legal action, the lack of a regulatory framework, physicians being pressured by (mostly uninformed) family members and physicians’ personal motives were reported as important reasons behind providing futile medical care. The nursing professional’s role as a participant in decisions on futile care and as a mediator between physicians and patients (and family members) was highlighted. Furthermore, the patient’s role in decisions on futile care was prioritized. The patient’s effort to keep themselves alive was also highlighted. This effort impacts nursing professionals’ willingness to provide care. Providing futile care is a major factor that negatively affects nursing professionals’ inner attitude towards performing their duties. Finally, the psychological benefits of providing futile medical care were highlighted, and the importance of the lack of adequately developed end-of-life care facilities in Greece was emphasized.

**Conclusions:**

These findings enforce our opinion that futile medical care should be conceptualized in the strict sense of the term, namely, as caring for a brain-dead individual or a patient in a medical condition whose continuation would most likely go against the patient’s presumed preference (strictly understood). Our findings were consistent with prior literature. However, we identified some issues that are of clinical importance.

## Background

Medical futility is a long-held concept. It was introduced in the 1980s as a distinct concept of bioethics, which, however, is not a well-defined concept. It is extremely difficult (if not unattainable) to precisely define medical futility. The American Medical Association Council on Ethical and Judicial Affairs is of the opinion that medical futility is “inherently a value-laden determination” and has stated that “a fully objective and concrete definition of futility is unattainable” [1]. It has been arguably stated that medical futility is “an elusive concept” [2], similar to other terms such as love and art [1]. While medical futility was initially strictly conceptualized based on evidence-based medical judgements, over the last decade, it has been broadly conceptualized based on not only medical but also value judgements [3]. Recently, consistent with previous literature [1], Katz stated that “futility in medicine has been defined as excessive medical intervention with very little prospect of altering the clinical outcome in a positive manner” [4]. Katz attempted to provide a broad definition and stated, “If treatments fail to release our patients from the preoccupation with the illness and do not allow them to pursue their life goals, then perhaps that treatment is futile” [4].

White et al. put it best by saying that “futile treatment has been commonly understood in two senses: first, the likelihood that treatment will confer patient benefit is unacceptably low (quantitative futility); second, the quality of the resulting patient benefit is unacceptably low (qualitative futility)” [5]. Note, however, that it has been argued that the concept of medical futility (broadly understood) comprises the following four aspects: a) the chance of success, namely, achieving the desirable goals, is extremely low; b) there is consent of patients or their surrogates; c) the patient’s quality of life is poor; and d) the consumption of medical resources is not proportional to the expected benefits. Every single health professional may make a different judgement with regard to these aspects when considering the situation of a particular patient [1].

Nursing professionals are frequently confronted with situations where they believe that providing further treatment is no longer beneficial to the patient without imposing extra suffering to them. By profession, nursing professionals are very closely involved in providing direct care to patients who may be in the final stage of life and especially near death. They may be in prolonged, continuous, and intense contact with these patients. The nursing professional’s role is related to (both physical and narrative) proximity, namely, nearness to the patient’s body while understanding his or her story [6]. At least in many developed countries, the majority of deaths occur in hospital settings against patients’ wishes. As nursing personnel spend the most time with the patient, and the role of nursing professionals in end-of-life decision-making remains unclear [7], further investigation is needed to determine more about their perceptions of “futile treatment”.

Medical futility remains a key priority research topic for clinical ethics. In Greece, little research has been conducted on this topic. This study aimed to contribute to filling this knowledge gap. We conducted qualitative research involving in-depth interviews focused on investigating the medical futility experiences of nurses with a long history of caring for severely ill or terminally ill patients.

## Methodological aspects

### Objective

The present work was formatted as a prospective qualitative research study centred on exploring the descriptions of nursing professionals’ lived experiences and attitudes towards futile medical care. Data were collected through semistructured in-depth interviews conducted in person (face-to-face) with 16 experienced nursing professionals between December 2020 and May 2021.

We became interested in the research topic stated in the title because of our previous experience with clinical practice. Medical futility is an existential and challenging experience that necessarily has subjective aspects, which patients and families go through by themselves. Therefore, the main goal of this study was to provide support in getting the patient’s voice heard by way of getting the nurses’ voice heard and ultimately promote a patient-centred approach in the context of end-of-life clinical practice. As the futility concept is relative to the desired outcome, in this study, futility was conceptualized in regard to the purpose of promoting a culture of caring for patients in a way that is valuable and meaningful to the individual patient. From this perspective, medical treatment is futile for the purpose of providing a benefit (regarded as such in the context of patient-centred care) when it fails to allow a patient to live for a period of time and at the same time be respectful of and responsive to individual patient values, preferences and desires.

### Research questions

The primary research question that defined the focus of this study was as follows:

What are experienced nursing professionals’ lived experiences with regard to medical futility in Greece?

The secondary research question was as follows:

What are the factors (if any) that can cause experienced nursing professionals to consider a medical intervention to be futile?

### Research team

PV is an associate professor of medical ethics. AT conducted the interviews. She has a degree in nursing and a degree in law. She also has a master’s degree in extracorporeal circulation, and at the time of the study, she was pursuing a postgraduate degree in ICUs (expecting to graduate) and a postgraduate degree in bioethics. AKT is a professor of experimental surgery.

### Study design

#### Theoretical framework

Thematic analysis was selected as the methodological orientation to underpin the study.

#### Participant selection

Purposive sampling was used to identify experienced nursing professionals who potentially had professional lived experiences with medical care considered futile. The participants represented a wide range of ages and previous work experience in nursing. All the participants had graduated from university nursing schools. Thirteen out of the 16 participants in this study possessed a master’s degree, and one participant possessed a doctoral degree. Initially, we approached nursing professionals who might meet our inclusion criteria using the interviewer’s (AT) personal contacts. Potential participants were approached in person, by phone or by email and then contacted by phone to schedule an interview. None of the potential participants refused to participate or dropped out of the study. Recruitment continued from December 2020 through May 2021 and ultimately reached a total of sixteen participants. After first contact, all the individuals were told that the purpose of the study was to better understand the medical futility experience of nursing professionals in Greece and that the interview was expected to take between 30 and 60 min to complete. After agreeing to participate, the participants received a brief explanation of the objectives and policies regarding anonymity, voluntary participation and confidentiality of the study.

#### Setting

The interviews were conducted in neutral places of the participant’s choice. All interviews were held in quiet places with a comfortable environment. As phenomenological researchers, we were interested in describing the participants’ experiences while maintaining a natural (normal, unreflective and effortless) attitude. No one aside from the participant and interviewer was present at the interviews.

The interviewer did not hold any strong views about medical ethics and remained neutral on issues that were discussed with the interviewees. From this perspective, the interviewer did her utmost to not ask leading questions and to not interrupt the interviewee while they were speaking or disturb them while they were remaining silent. Moreover, the interviewer placed great weight on establishing a rapport between herself and the informants. The interviewer was particularly emotionally challenged, as she is a nurse with increased awareness of the topic of interest. As we aimed to have a neutral attitude, we ensured that this would be achieved by talking with each other about our thoughts and potential biases. The content and focus of these discussions were on leaving the interviewer sufficiently emotionally prepared for the information revealed in the interviews. The interviewer did not speak too much and thus allowed enough time for the interviewees.

#### Description of the sample

The inclusion criteria for participation in the study were (1) living and working as a nursing professional for at least ten years in Greece and (2) being experienced in providing care for severely or critically ill patients for at least ten years. Furthermore, participants needed to be (3) a graduate of a university nursing school. We thought that nursing professionals who had graduated from university schools could obtain an in-depth understanding of futile care and better express their experiences and perceptions of it. The selected study participants (*N* = 16) were experienced nursing professionals with considerable work experience with caring for severely or critically ill patients. None of them had worked in settings where severely or critically ill patients were not included. Participants were diverse in terms of age, gender identity, work experience and educational background. The age of the participants ranged from 30 to 52 years, with the majority being between 39 and 52 years. The mean (standard deviation, SD) age of the participants was 41 (SD = 6) years. The years of previous work experience ranged from 10 to 29, with the majority being between 14 and 29 years. The mean (SD) previous work experience of the participants was 17.5 (SD = 6) years. All the participants resided in Athens. The participant characteristics are presented in Table 1.

**Table 1:** Participant characteristics

Participant	Work experience	Age	Gender
P1	20	48	Male
P2	29	52	Female
P3	18	43	Female
P4	22	41	Female
P5	25	45	Female
P6	20	47	Male
P7	10	36	Female
P8	10	39	Male
P9	12	36	Female
P10	10	33	Female
P11	14	37	Female
P12	23	41	Female
P13	16	40	Female
P14	18	41	Female
P15	24	50	Female
P16	10	32	Female

#### Data collection

The interviews were conducted face-to-face. The development of the interview guide was based on a review of the relevant literature. As a first step, the interview guide was pilot tested. The guide was slightly refined based on the initial results from a few interviews to allow the participants to better understand the specific issues being asked in the questions. Then, we developed an informal grouping of topics and questions that the interviewer could ask in different ways for different participants. The interview guide covered a number of topics that aimed to capture a wide range of the participants’ lived experiences. These topics were related to a) the factors that can cause nursing professionals to deem an intervention as futile care, b) the reasons behind providing futile medical care, and c) the consequences of providing futile medical care. The participants were encouraged to expand upon the examined topics. They were asked broad questions and encouraged to respond in a conversational way to express themselves. The interviews were semistructured and started with questions such as “What does futile medical care mean to you?” (*a grand tour question to make the participant comfortable*), “What would or did motivate you or other health care professionals to deem medical treatment as futile?”, “What *factors would contribute* to *your patient experiencing* a ‘*good death*’ or a ‘dignified death’*?”*, “What is it like to be a nursing professional caring for a patient who is receiving futile medical care?”, “Can you please describe in detail what were your responses to providing futile medical care?”, “How do you think others perceive providing futile care?”, “What does dying a ‘good and dignified death’ mean to you?” and “What do you know about other health professionals experiences or attitudes towards providing futile care?”. Additional questions were asked to elicit more detailed explanations and identify the essential themes of nursing professionals’ perceptions of futile medical care. For instance, two explanatory questions focusing on further exploring the answer given for the *grand tour question* in a more detailed and deeper manner were the following: “Irrespective of the physicians’ opinion, what (and why) would *you* (if you) consider to be provision of futile care or treatment to a terminally ill patient who, however, is not yet determined to be brain dead?” and “In your opinion what is the importance of defining futile?”

The interviewer audio-recorded the interviews to collect the data. In addition, field notes were made after the interview to record nonverbal behaviour patterns, as well as procedural and contextual aspects of the interviews, which enabled deeper and contextual critical reflection on the data collected. The interviews lasted from 38 min to 55 min each (mean 44 min). We stopped data collection when we believed data saturation was reached, namely, when no additional information was obtained from further interviews. The interview transcripts were not returned to the participants for their comments and/or corrections.

#### Data analysis

Qualitative data were analysed using thematic content analysis [8]. Verbatim transcription of the audio-recorded narratives was performed. We followed Gibbs’ advice on demonstrating qualitative reliability [9]. Using this perspective, we carefully examined, verified and repeatedly read the transcripts to obtain a good sense of the participants’ narratives [8]. We constantly compared the data (as described by Patton [10]) to ensure that the codes were used consistently. The data obtained from the interviewees were thematically categorized and analysed. Open coding was used to identify quotations related to our research questions. Three data coders coded the data. We did not provide a description of the coding tree. After summarizing these quotations in notes, we grouped the phrases that reflected the same context to form categories and subcategories that might represent starting points for the results of the study. Then, the transcripts were reread and constantly compared with the list of categories and subcategories to identify further phrases in transcripts that might help address the research questions. Therefore, we strived to capture and investigate in depth all aspects of the participants’ narratives related to the research goal. Moreover, we coordinated communication and shared analyses.

A data management software program (NVIVO, 2015) was used to secure and further refine the systematic character of the analysis. The participants did not provide feedback on the findings. Participants’ quotations are presented to illustrate the themes and findings. Reflexive thinking was used throughout the research process to reduce unintentional personal bias. Each of us engaged with the other researchers to limit the amount of research bias.

### Ethical considerations

The interviews were conducted in neutral places of the participant’s choice, thereby ensuring privacy and confidentiality and minimizing environmental impact. We adhered to the ethical principles of anonymity, voluntary participation and confidentiality. The participants’ anonymity and confidentiality were maintained throughout the study; to preserve their anonymity, pseudonyms were used to describe participants in this study, and the interviews were registered and stored in a strictly confidential fashion. The study and consent procedure were approved by the ethics committee affiliated with Aristotle University of Thessaloniki, Faculty of Health Sciences, Department of Medicine (No: 3.392/22-12-2020). In addition, we confirmed that **all methods were performed in accordance with the relevant guidelines and regulations.**

## Results

Participants’ perceptions of futile care varied depending on the patient’s condition and the participants’ personal perspective of the patient’s condition. The perceptions of the participants in our study on medical futility are related to the moral values of respecting patient wishes, easing suffering, maintaining truthfulness and distributing scarce health care resources appropriately.

### The concept of “futile medical care”

While participants might each have a familiar sense of what we mean by invoking the term “futile medical care”, the term was not explicitly defined. Several participants in this study have shown cautious attitudes towards labelling medical treatment as futile. Our data analysis suggests that this is because these participants not only realized the difficulty of providing a clear definition of the entity “futile treatment” but were also hesitant as to whether they could view life-sustaining medical treatment as futile. However, participants viewed the concept of “futile treatment” as familiar. They appeared to have a sense of the term “futile medical care”. Some participants viewed the concept of “futile medical care” as quantitative futility, while some participants viewed it as qualitative futility, and some participants attempted a mixed approach.

More precisely, 7 out of the 16 participants in this study (P4, P7, P9, P11, P13, P15 and P16) felt that providing medical care to patients with no life expectancy is providing futile care. However, they did not attempt further to define the term “life expectancy”. Please note that especially important things can be said or experienced in the last hours of a patient’s life. Two out of the 16 participants (P8 and P12) felt that medical care that cannot improve the patient’s quality of life is futile, without attempting to further define the term “quality of life” in the context of end-of-life care. Three participants (P3, P5 and P6) said that futile care is ineffective care, without attempting to further define what is meant by the term “ineffective”. Participant P14 viewed as futile any treatment that proves harmful to the patient in the sense that it imposes extra pain or suffering on him or her.

Interestingly, several participants (P1, P2, P10 and P14) explicitly expressed their concerns about labelling medical care as futile. Participant P2 said that there were difficulties in defining the concept of “futile medical care”. More precisely, Participant P2 said, “*It is difficult for someone to determine situations that could cause health professionals to consider a medical intervention as futile*”. She said that she had learned from her professional experience that many elderly people are discharged alive from intensive care units. Furthermore, she attempted to provide a definition based on survival rates and quality of life. Participant P1 reported thinking in a similar vein. In addition, the following interview quotes are indicative of the difficulty of providing (or having a reluctance to provide) a clear definition of the concept of “futile medical care”. Participant P1 suggests that “*the concept of* ‘*futile medical care’ needs to be discussed by an international scientific community*”. Participant P6 suggests that “*the determination of medical futility can only be made within the context of clinical situations, which however, should be clearly outlined*”. Participant P13 said, “*There is [the concept of] “futile medical care” but … I don’t know [if it is morally right to accept it] …*” . Participant P10 said, “… *a medical intervention … perhaps … must never be labelled futile …”* . Participants P1 and P14 declared, “*I do not like the term futile medical care*” and “*The concept futile medical care is a hard concept*”, respectively.

### Patient reliance on machines – technological dependency

Participants P5 and P6 believed that mechanical support should only be provided to patients who have a chance of recovery from being dependent on such support. Interestingly, however, some participants expressed strong reservations on this view. Participant P10 emphasized that “*technology by itself cannot make it futile to further provide medical treatment*”. She said that medical treatment can by no means be considered futile if there is verbal or nonverbal communication (i.e., through eye contact) of the patient. Participant P11 clearly declared that providing further medical care to a brain-dead patient should be considered futile. “*However*”, she said, “*I do not know if the provision of life-sustaining treatment to a patient in a persistent vegetative situation might be thought of as futile*”.

### Reasons behind providing futile medical care

Fear of legal action, the lack of a regulatory framework, physicians being pressured by (mostly uninformed) family members and physicians’ personal motives were reported as being important reasons behind providing futile medical care.

Fear of legal action was a factor that emerged from our data analysis as a significant reason behind providing futile medical care. This was a recurrent finding throughout the narratives of the vast majority of participants (14 out of the 16 participants in our study). Only two participants (P2 and P7) believed (even though not wholeheartedly, i.e., using the expression “*rather not*”) that the fear of legal action was not a significant reason behind providing futile medical care. Furthermore, physicians being pressured by family members into providing futile medical care (P3, P4, P14, P15, and P16) and the lack of a clear regulatory framework for futile medical care in the country (P1, P4, and P14) were reported as driving forces for the provision of futile medical care through intensifying health professionals’ feeling of fear of legal action. A couple of participants (P5 and P8) reported that in some hospitals where there have been developed documents (according to protocols, regarding the provision of futile medical care) that are to be signed by the patients or their representatives (i.e., family members), the fears of lawsuits have become limited.

Moreover, some participants believed that the physicians were the drivers of futile medical care. Participant P2 said that the physicians were the drivers of futile medical care because of their emotions or their personal motives, such as the egoistic motive for “*carrying it off*”, namely, coming out on top. She said that physicians are not afraid of lawsuits. Participant P7 said that “*psychological and emotional factors are more significant reasons behind providing futile medical care than the fear over encountering legal problems”*. At any rate, it should be highlighted that many participants were of the opinion that physicians often provide futile medical care to their patients.

Participant P3, who had long previous work experience in intensive care units, wondered why many futile treatments are provided in ICUs where the probability of taking any legal action against health professionals is very low. ICUs are specialized facilities where the staff’s actions are less than transparent and open to others.

Interestingly, three participants argued in favour of providing futile medical care, at least under particular circumstances. They reported their previous lived experiences during their long career history (more than twenty years) as nursing professionals. Participant P2 reported a case of a patient in a critical situation. There were pressures made by nursing personnel to the physician to consider medical care futile and to “*let him leave*”. However, the physician’s persistence in providing futile medical care resulted in the patient being discharged from the intensive care unit in a “*good level of conscience*”. Experienced nursing professional P1 said that “*while the patient was expected to live for a very short period of time, ultimately, he lived much longer*”. Participant P5 reported that he had witnessed the case of a child in the end-of-life phase. The medical team decided to consider the provided care to be futile and to interrupt it. The child died during seizures. The participant who witnessed that scene was emotionally changed for the rest of her life.

Interestingly, Participant P2 emphasized the lack of facilities that provide end-of-life care. She said that while many patients who have been treated in intensive care units are ranked as severely disable at discharge, further treatment of these patients may be erroneously labelled futile although such treatment might be beneficial and valuable to them (might improve their quality of life) if provided in a palliative facility or at the patients’ own home. Patients in such situations cannot be treated sufficiently in an understaffed classic open hospital ward.

### Consumption of considerable resources

Furthermore, the wastage of money and the consumption of considerable resources are reasons for labelling medical care as futile. The vast majority of participants declared that spending money and the consumption of resources should be factored in when considering the provision of a medical treatment that can be labelled futile, especially in the time of the Greek financial crisis, namely, over the last decade, when the available resources have been limited (P9 and P14). Note, however, that most participants appeared to not wholeheartedly support this consideration. The expression “*even though it seems unfair*” was recurrent in the interview data (P3, P4, P8, and P14), especially because the patients had paid their health insurance contributions (P6 and P11). Participants P2 and P3 said that we are obliged to take into account the costs, especially in regard to providing medical care in intensive care units where the consumption of resources is considerable. Two participants (P11 and P12) expressed some clear reluctance to accept the role of costs in end-of-life decision-making. Participant P1 expressed his clear negative attitude towards the suggestion that costs should be taken into account when making end-of-life decisions. In contrast, Participants P2 and P16 provided wholehearted support for this consideration, without reservations.

The high costs of futile medical care delay accessibility to health care resources for patients who have life expectancy (P9, P10, P12 and P14). Participant P15 clearly determined the term “life expectancy” when considering the role of costs in labelling medical care as futile. She said, “*If a patient has a chance to survive, the costs should not be taken into account*”, thereby implying that even a very low survival rate may be important. Participant P7 said that we should not take into account the costs in regard to facilitating a dignified death. She said, “*Ensuring a dignified death is the only meaningful thing we can offer to these people [patients nearing the end of life]*”. Indicative of the participants’ half-hearted attitudes towards accepting the role of costs in deciding about futile medical care was the speech of Participant P10, who was of the opinion that the money saved by avoiding or stopping futile medical care could be better spent creating the most advantageous conditions possible, such as “*providing better psychological support or palliative care … improving the atmosphere in the patient room, i.e., by painting the walls … making the patient laugh* …” However, the participant found this suggestion impracticable.

Participants P13 and P15 declared that physicians often do not consider the costs despite (health care administrator’s) recommendations to the contrary (P13).

### Who is the decider? The nursing professionals’ role in *deciding on* the *futility* of a certain *treatment*

In this study, the nursing professional’s role as a participant in decisions regarding futile care and as a mediator between physicians and patients (and family members) is highlighted. Furthermore, the patient’s role in decisions on futile care is prioritized.

Almost half of the participants suggested that the end-of-life medical decision process should incorporate the patient (prioritizing their preferences) and the attending physician (P2, P4, P5, P7, P8, P9, and P14). Participant P3 was of the view that physicians should make unilateral end-of-life decisions after having listened to the nursing personnel. However, almost half of the participants suggested that the end-of-life medical decision process should always incorporate all key stakeholders, including nursing professionals and family members (P6, P7, P10, P12, P15, and P16). Importantly, Participant P4 stressed that a person who is in a long-term proximity relationship with the patient (“*the closest person to the patient*”) should be considered a close family member.

Participants recognized that nursing professionals are frequently not involved in the end-of-life decision-making process (P7) and that therefore, they feel abandoned and powerless (P14). Participants wanted to be more involved in end-of-life decisions (P3, P10, P11, and P13) because nursing professionals spend much more time with the patient than do physicians. Participant P13 said, “*Nursing professionals and patients rub along together*” and considered that nursing professionals should play a leading role in end-of-life decision-making. Importantly, P14 said that nursing professionals understand the patients’ values and preferences, and hence, they are ideally positioned to act as mediators between physicians and patients (and family members). Participant P7 said, “*Nursing professionals are included in end-of-life decisions, although to a limited extent*” and emphasized the need for good communication between health professionals for nursing professionals to be consistently included in end-of-life decision-making processes. Some participants considered it inappropriate for family members to be included in end-of-life decision-making processes because pressures from them are often a driving force for the provision of medically futile care (P2, P8, and P14).

### Provision of information to family members

From our data analysis, it emerged that patient family members often keep (false) hopes alive due to a lack of information and knowledge. It is so difficult for them to accept that the death of a loved one is inevitable, and they believe in a miracle. For instance, Participant P4 (who had 22 years of previous work experience) said, “*Nobody is wired to accept that death happens …”* . Family members may become quarrelsome and imply that in the case of negative development, they might take legal recourse against the attending physicians (P4). It was emphasized that the bonds between family members are particularly strong in Greece (P7). Participants highlighted that in the Greek clinical context, patients and family members lack information about end-of-life issues (P1, P4, P7 and P14). It has been stated that well-informed family members (especially when the information provided is accompanied by psychological support) are expected to put less pressure on physicians by trying to cause them to provide futile medical care (P10). Participant P8 suggested that public education should focus on the specific and limited role of intensive care units, as well as the option of palliative care, to prevent the general public from having unrealistic expectations. Interestingly, two participants (P1 and P10) highlighted the psychological benefits of providing futile medical care mainly due to offering (false) hopes. Participant P1 (who had 20 years of previous work experience) said, “*Let’s see it in a holistic way … providing futile medical care may be psychologically beneficial, even if it may not be biologically beneficial … nobody knows how much grief and death cost in our inner world*.”

### Participants’ personal responses to situations where *futile care* is provided

Our data analysis showed that providing futile care is a major factor that negatively affects nursing professionals’ inner attitude towards performing their duties. While participants developed resilience-promoting strategies to deal with providing futile medical care, they emphasized the negative impact of providing futile care on their inner world. A tension that exists between their inner world and outer world emerged from our data analysis.

Interestingly, participants normalized their negative experiences of providing futile care, stating that they were performing their duties to the best of their ability and with respect for their professional standards. However, in apparent contradiction to these assertions, they expressed concerns about their inner attitude towards caring for patients receiving futile medical treatment and emphasized the emotional nature of their duties. The open-ended questions permitted nurses to specify situations of futile care, which they regarded as morally distressing.

Six out of the 16 participants in this study (P2, P5, P6, P7, P11, and P12) declared that providing futile medical care does not affect their behaviour towards their patients and family members. Interestingly, Participant P6 said that he takes care not to change his behaviour towards his patients because “*patients nearing the end of life understand*”. Participant P7 said that she takes care not to change her behaviour towards her patients (nearing the end of life) because she “*must ensure good quality of life for her patients right to the end*”.

Notwithstanding, ten participants declared that the negative emotions they experienced because of providing futile medical care negatively affected their attitude and behaviour towards their patients. Participant P13 said*, “When you know that medical care is futile, you are not in the mood to ‘run’, to communicate with the patient … when you feel that your efforts are not ‘paid off’ [on an emotional level], you feel that you are performing drudgery*”. Participant P3 declared, “*I may not be on-task … I may make omissions to provide the complete treatment to the patient*”. Participant P10 said, *“If there is a sense of vanity in the air, nursing professionals may not be in a good mood and become less communicative*”. However, Participant P10 said, “*If the patient does not express (by any means) a strong desire to struggle to save his or her own life, then nursing professionals are working in a dominant atmosphere of futility*.” In the same vein was Participant P14, while Participant P8 declared that nursing professionals distance themselves from their patients.

Participants P2, P5, P6, P7 and P11 said that they are able to get their emotional reactions under control; i.e., Participant P2 said, “*I am often emotionally affected but … I can turn off the switch …”* .

Regarding the emotional experiences of caring for patients who receive futile medical care, many participants declared that they often experience sadness and grief (P4, P7, P9 and P14). Interestingly, some participants reported that they often feel anger or become stubborn for various reasons. Importantly, it has been reported that they feel that providing futile care saps their energy.

Participant P12 said that in such situations, she often becomes stubborn about ensuring that patients and families experience a “good” and dignified death. She said, “*When providing futile care, you have to show greater respect for the patient’s body.*” Participant P9 felt that providing futile care sapped her energy. She said that doing so “*wastes my energy, which is something needed by other patients*.” In a similar vein was Participant P14, who declared that providing futile care is “soul-sucking.” The participant said, “*I feel so angry with myself when I am forced to give false hopes to a sick person who looks at my eyes and say things that she would never say to her family.*” Participant 4 had said almost exactly the same thing. Participant P16 said, “*We get angry because of the failure of our efforts to succeed … we are emotionally charged*.” Importantly, Participant P1 said, “*There are negative emotions in the inner world of nursing professionals, which will never be externalized* …” . Interestingly, Participant P9 said, “*I feel anger because providing futile care wastes my energy … which is something that other patients need …”* .

### The concept of a “good or dignified death”

All participants took a clear position on a “good and dignified death”. Interestingly, from our interview data analysis, a list of criteria emerged for assessing the patients’ quality of life and labelling the dying process as undignified, which, when identified in clinical situations involving medical treatment, the treatment might be perceived as futile.

A range of various and distinct features of a “good and dignified death” emerged from our data analysis. Some of them appeared as recurrent findings. These features can be roughly categorized into four categories. Features revolving around a) pain and suffering, b) treating patients with respect, c) the appearance and image of the patient body, and d) the interaction and interrelation between patients and their relatives emerged.

Among the features of a good and dignified death have been cited 1) the patient’s pain and suffering (P5, P6, P7, P10, P13, P14 and P16), 2) the enforced continuation of a patient’s (full suffering) (P13 and P15), 3) treating the patient not as a person but as a medical event, (P14), 4) a patient dying without being prepared for his or her own death (P1), 5) the lack of respect for a patient’s person or personality (P2, P3 and P14), 6) the patient’s inability to determine how he or she will die (P8), 7) the lack of cleanliness (P4, P7 and P14), 8) the patient being incapable of self-serving and being completely dependent on others (P8, and P9), 8) the patient’s feelings of humiliation and powerlessness (P14), 9) the patient’s unmet actual (P14) or presumed preferences, i.e., not ensuring religious patients have the opportunity to take holy communion (P2), 10) the patient feeling incapable of keeping pace with what is meant by the term “human being” or “the human condition” (P3), and 11) the patient feeling as though they are imposing on others (P8).

Furthermore, many participants have recurrently placed considerable emphasis on two main aspects of “bad or undignified death” that deserve particular attention, namely, the appearance and image of the patient’s body and the interaction and interrelation between patients and their relatives. A number of situations have been reported by several participants (experienced nursing professionals) to be features of what is meant by the term a “bad or undignified death”.

The appearance and image of the patient body regarding the patient’s appearance is a) taken as it truly is, b) as it is perceived to be by the patient, or c) as it is seen through others’ eyes. The patient dies an undignified death if a) his or her body is “melting” (P11), b) he or she is getting bed sores (especially if the bed sores are neglected) (P7 and P13), c) the patient’s body has changed and lost its shape (P11), the patient’s self-image has degenerated into a decomposition image (P3), d) “*150 sachets*” are attached to the patient’s body (P4), e) a Levin feeding tube is inserted into the patient’s body (P4), f) the patient undergoes many painful venipunctures daily, while nearing his or her inevitable end of life (P12), g) the patient is not capable of self-serving (especially defecating or urinating) (P8), h) the patient arouses pity for him/herself (P5 and P9), the patient begs to die (P5), i) the patient is not capable of speaking or communicating with others (P8), j) the patient is getting naked often (P4), and h) the patient would feel sad if he or she could look at him/herself in the mirror (P5).

The interaction and interrelation between patients and their relatives is a factor that has been reported to be an important aspect of a “bad and undignified death”. It has been said that dying a death in isolation, in a “cold” hospital environment, is not dying a “good and dignified death”; rather, dying in the home environment helps the patient die a “good death” (P4 and P9). Dying a dignified death means dying surrounded by family and loved ones, communicating and being in contact with them, i.e., clutching the patient’s hand tightly or caressing the patient’s head (P2 and P4), and giving one’s love to the patient (P14), thus making the patient feel that they are not feel alone (P2, P4 and P7) and that, as “*patient and family (or other loved ones) get through hard times together*” (P7), the patient has people around him or her to share his or her fears (P4). Family members must create an optimistic environment (P10) where there is no mourning (P14). Participant P2, who has had much experience in the specific field of intensive care medicine, said, “*Patients under sedation need human contact*” and “*You never know what a patient under sedation can understand*”. Importantly, the participant added, “*I had a habit of caressing the head of a child who was in sedation … when she woke up, she told me that she kept seeing me in her dreams caressing her head.*”

## Discussion

### The concept of “futile medical care”

The participants arguably had difficulties in defining medical futility in a clear way. Lantos et al. long ago put it best in saying that medical futility is not an objective entity and “must be determined in the light of the subjective views and goals of patients” [2]. Furthermore, some participants perceived medical futility as rather quantitative futility, with some others perceiving it as rather qualitative futility.

Futile treatment has been commonly understood in two aspects: qualitative and quantitative [5]. Some experts explain futile care only in terms of patient survival, while others consider quality of life, in addition to patient survival [11]. In several previous studies, nursing professionals described futile medical care as ineffective care [12], failing to improve patient survival rates regarding the overall outcome benefit to the patient’s health [5] or prolonging suffering without providing “reasonable hope” for a patient to recover to “a state of relative independence or be interactive with their environment” [13] while using “considerable resources” [13]. Such medical care fails to improve the patient’s quality of life while prolonging his or her suffering [13]. Interestingly, while White et al. found that futility is a familiar term with which doctors readily engage, they noticed “a high degree of variability in how this definition is applied in the clinical setting, reflecting the qualitative nature of patient benefit” [5].

Factors cited in the literature as factors that can cause nursing professionals to consider a medical intervention as futile treatment revolve around the following key themes: ineffective treatment, prolonging or imposing extra suffering, and the considerable wastage of resources that might truly benefit other patients [12, 14, 15]. Note, however, that scholars fail to quantify or further specify how to determine these terms.

In contrast, the lack of *healthcare structures* in *palliative medicine and hospice care may be important reasons behind* labelling medical care as futile [16]. The further treatment of some patients may be erroneously labelled futile despite the fact that it might be beneficial to them if provided in a palliative facility or at the patients’ own home.

### Patient reliance on machines - technological dependency

The participants’ half-hearted attitude towards labelling the life-term reliance on machines as futile medical care is consistent with their general hesitant attitude towards considering a medical intervention as futile care (as presented in other parts of the Results section).

We feel that the experienced nursing professionals’ attitudes are intuitively and morally justified. We are of the opinion that futile medical care should be conceptualized in the strict sense of the term, namely, as caring for a brain-dead individual or a patient in a medical condition, whose continuation would most likely go against the patient’s presumed preference (strictly understood).

### The reasons behind providing futile care

The most important reasons behind providing futile medical treatments that have been cited in the literature are “patients’/family members’ request and persistence”, “health care professionals’ personal emotions, beliefs, and attitudes”, and “organizational factors and fear over getting involved in medical litigation” [13, 15, 16]. It has been argued in the literature that family members (more particularly family caregivers) demonstrate stronger insistence for providing futile medical care than patients themselves [17]. Family members insist on providing futile care because they have unrealistically high expectations because they are not well informed by physicians (who may be unwilling or unskilled in discussing end-of-life issues) about the underlying conditions [16, 18], have difficulty accepting the reality of an imminent death, and fear “loneliness and being neglected” [16]. Thus, communication between health professionals and patients or patients’ family members is essential for making better end-of-life decisions and judgements about futile treatments [7]. It has been stated that “open and honest communication with patients and their families about treatment efficacy, expectations regarding outcomes, and other related considerations may help limit some futile treatments” [1]. Moreover, it has also been argued that efforts to limit futile medical care should include public information about the role of intensive care units (ICUs) and palliative care [18].

At any rate, a lack of legal support has been cited as a reason for complying with family members’ insistence on providing futile care [13]. Surprisingly, it has been suggested that physicians may be the drivers of futile medical care because of their personal motives, such as “prognostic uncertainty or their perception of healing as the core purpose of medicine, and the so-called treatment imperative” [16]. Note that Willmot et al. found that physicians believe that “a range of factors contribute to the provision of futile treatment” [19].

In contrast, providing futile care may help family members visit the patient and be with them during the last hours of their lives [16]. Furthermore, it has been argued that “ethically and legally doctors are not obliged to provide futile treatment to patients, even if the patient or their proxies are prepared to pay for it. However, it may be justified where such treatment is harmless and has a placebo effect” [20].

### Who is the decider? The nursing professionals’ role in *deciding on* the *futility* of a certain *treatment*

Physicians are often the primary decision-makers who make end-of-life decisions unilaterally. Nursing personnel are expected to follow these decisions. In some studies, participants (nursing professionals) have agreed that physicians are the leaders of the decision-making process [7]. Some of the participants in our study thought similarly. Note, however, that this is a completely paternalistic and morally unacceptable approach. End-of-life decisions are a shared decision-making process in which all stakeholders (i.e., patients, relatives and health care teams, ethicists, spiritual care counsellors) must be involved [13, 18, 21]. The end-of-life decision is a difficult decision that is influenced by many individual patient factors [7, 21].

Nursing professionals are the main caregivers who spend most of their time near the patient and are in close physical and narrative proximity to them, thereby encountering their particularities [6]. However, the literature reports the underrepresentation of nursing professionals in end-of-life decisions [7, 22, 23].

Nursing professionals are arguably motivated by a rigid *desire* that attending physicians should *listen* to them [22]. More generally, nursing professionals have highlighted the need for better interprofessional relationships in regard to end-of-life decisions [23]. Note, however, that it is argued that physicians often recognize the role of senior and experienced nursing professionals and include them in end-of-life decision-making processes [24].

### Nursing professionals’ responses to situations where *futile care* is provided

Among the most important factors that cause nursing professionals’ moral distress are a) witnessing unnecessary suffering and providing unwarranted and overly aggressive treatment [25, 26], b) disregarding a patient’s preferences [25], c) a shortage of resources and inappropriate resource allocation and utilization [25], and d) seeing a patient die without having an opportunity to say farewell to their relatives and acquaintances [27]. All these factors are consistent with our findings. Furthermore, as nursing professionals are in close physical and narrative proximity to end-of-life patients, they may experience moral distress and a desire “to escape their responsibility” [6].

As a means of defence (i.e., to minimize their own stress), nursing professionals who are experiencing moral distress may distance themselves from a patient and provide poor quality care [28]. However, most of the participants in the current study asserted that they manage to prevent negative effects on patient care in such situations. To appear professional, nurses may use mechanisms such as the downregulation of emotions [29]. In line with Participant P9 in our study, a participant in a previous qualitative research study felt that providing futile care saps her energy [30].

### The concept of “good and dignified” death

As presented in the Results section, many participants specified various and distinct situations that, in their view, make the dying process an undignified process and therefore could cause them to view medical treatment as futile. In our opinion, continuing these situations is most likely to be in conflict with the presumed preferences of the patient.

The concept of a good death is highly individual; thus, the patient’s preferences should take precedence [23, 31, 32], and the patient’s integrity should be protected [32]. Dignity is perceived by nurses as a multidimensional entity [33]. Efstathiou and Ives put it best by saying that “dignity is both inward and outward looking, concerned both about how the death is or would be experienced by the dying patient and how it is perceived and experienced by others. This was illustrated through symptom control, physical cleanliness and removal of technical apparatus” [34]. The concept of a “dignified death” or “good death” appears to be connected to maintaining dignity for the dying patient and achieving a less medicalised death [34]. Maintaining dignity for the dying patient is an essential aspect of end-of-life health care. In that regard, it is noteworthy that Parmryd et al. found that nursing professionals in critical care settings put forth every effort to protect patient integrity [32]. However, a “dignified death” is an abstract concept that is difficult to define. Within this study, a “dignified death” was often talked about in connection with acts such as symptom control and relief from suffering, physical cleanliness and the good appearance of the patient’s body, the removal of technical apparatus and providing patients with time to say farewell to their relatives and acquaintances. Relief from pain and suffering and the provision of physical care for dying patients are essential for a dignified death [35]. Note, however, that “suffering” is a “rarely explicitly defined” concept, which is used to refer to a “negative, subjective experience that goes beyond the experience of pain” [36]. The physical presence of family members is regarded as essential during a good death [22]. Being surrounded by and communicating with family members is essential for dying a “good death.”

Authors have argued that making the dying patient look clean and fresh reinforces dignity [35]. The poor appearance of the patient may result in separating the patient from the family, whereas a good appearance may contribute to “reconnecting” the patient with their family, namely, promoting a good death [37]. Note that in the literature, it is argued that body image impacts patient care and “influences a number of medical-related outcomes” [38]. Further investigation is necessary to explore how the deteriorating physical appearance of the patient (who already looks dead, and their body appears to be rotting) factors into the futility, as determined by nurses [39].

### Limitations

First, although we put great effort into conducting a selection process that was free from biases, potential self-selection bias cannot be ruled out. Those who were particularly interested in the topic of research are more likely to have responded to our call for research participation. Second, our sample size was small. However, it was similar to that found in other qualitative studies. Although saturation was reached, further research should explore these topics in more depth. Third, a limitation of this study is that only participants working as nursing professionals at hospitals in Athens were interviewed. However, the results of this study provide valuable insight into experienced caring for patients near their end of life and participants’ perceptions of futile care. While many nursing professionals in the country are working under similar circumstances, we argue that the results are transferrable to similar contexts. Fourth, a limitation of this study is the fact that the data were only collected via face-to-face interviews and field notes. Other methods of data collection relying on other methods, including focus groups, can enrich the findings of a similar qualitative work of research. Furthermore, recall bias may have occurred, at least with regard to certain findings. Finally, any qualitative interview study is prone to interviewer and researcher bias.

## Conclusions

From our data analysis, a nursing professionals’ half-hearted acceptance of futile medical care has emerged. This attitude is consistent with other findings of this study, such as those highlighting the positive aspects of futile medical care and the participants’ reluctance to label medical care as futile for the sake of saving costs and resources. Interestingly, within this study, a number of clinical situations emerged that best describe in detail what is meant by the term a “bad and undignified death”. These findings enforce our opinion that futile medical care should be conceptualized in the strictest sense of the term, namely, as caring for a brain-dead individual or a patient with a medical condition, whose continuation would most likely go against the patient’s presumed preference (strictly understood). Furthermore, for a great part, our findings were consistent with prior literature. However, we identified some nuances that are of clinical importance. Among these findings, the nursing professionals’ role as a mediator between physicians and patients (and family members) and the impact of patients’ (when nearing the end of life) expressed effort to live and their desire to live based on nursing professionals’ willingness to provide them with care are highlighted. Patients who are battling to keep themselves alive increase nursing professionals’ motivation to continue providing care for these individuals. In addition, the psychological benefits of providing futile medical care are highlighted, and the importance of the lack of adequately developed palliative care or care at home for patients nearing the end of life in the country is emphasized.

## Data Availability

The transcripts of the full interviews that were collected and qualitatively analysed in the current study are not available due to the ease with which study participants could be identified. The redacted transcripts used and analysed during the current study can be made available by the corresponding author on reasonable request.

## Abbreviations

ICU: Intensive care unit

## References

[1] Swetz KM, Burkle CM, Berge KH, Lanier WL (2014). Ten common questions (and their answers) on medical futility. Mayo Clin Proc.

[2] Lantos JD, Singer PA, Walker RM, Gramelspacher GP, Shapiro GR, Sanchez-Gonzalez MA, Stocking CB, Miles SH, Siegler M (1989). The illusion of futility in clinical practice. Am J Med.

[3] Hsu MY, Su SF, Chiang LY, Shih SJ, Chen YC (2018). The medical futility experience of nurses in caring for critically ill patients. J Nurs Res.

[4] Katz P (2020). When does treatment in Cancer care become futile?. Clin J Oncol Nurs.

[5] White B, Willmott L, Close E, Shepherd N, Gallois C, Parker MH, Winch S, Graves N, Callaway LK (2016). What does "futility" mean? An empirical study of doctors' perceptions. Med J Aust.

[6] Peter E, Liaschenko J (2004). Perils of proximity: a spatiotemporal analysis of moral distress and moral ambiguity. Nurs Inq.

[7] Flannery L, Ramjan LM, Peters K (2016). End-of-life decisions in the intensive care unit (ICU) - exploring the experiences of ICU nurses and doctors - a critical literature review. Aust Crit Care.

[8] Burnard P, Gill P, Stewart K, Treasure E, Chadwick B (2008). Analysing and presenting qualitative data. Br Dent J.

[9] Gibbs GR (2007). Thematic coding and categorizing, analyzing qualitative data.

[10] Patton MQ (2002). Two decades of developments in qualitative inquiry: a personal. Experiential Perspective Qual Soc Work.

[11] Rostami S, Jafari H (2016). NURSES' perceptions of futile medical care. Mater Sociomed.

[12] Aghabarary M, Nayeri ND (2016). Nurses' perceptions of futile care: a qualitative study. Holist Nurs Pract.

[13] Sibbald R, Downar J, Hawryluck L (2007). Perceptions of futile care among caregivers in intensive care units. CMAJ.

[14] Yekefallah L, Ashktorab T, Manoochehri H, Hamid AM (2015). Nurses' experiences of futile care at intensive care units: a phenomenological study. Glob J Health Sci.

[15] Aghabarary M, Dehghan NN (2016). Medical futility and its challenges: a review study. J Med Ethics Hist Med..

[16] Aghabarary M, Nayeri ND (2017). Reasons behind providing futile medical treatments in Iran. Nurs Ethics.

[17] Hwang H, Yang SJ, Jeong SY (2018). Preferences of older inpatients and their family caregivers for life-sustaining treatments in South Korea. Geriatr Nurs.

[18] Demir A, Sançar B, Yazgan E, Özcan S, Duyan V (2017). Intensive care and oncology nurses’ perceptions and experiences with ‘futile medical care’ and ‘principles of good death’. Turkish J Geriatr.

[19] Willmott L, White B, Gallois C, Parker M, Graves N, Winch S, Callaway LK, Shepherd N, Close E (2016). Reasons doctors provide futile treatment at the end of life: a qualitative study. J Med Ethics.

[20] McQuoid-Mason DJ (2017). Should doctors provide futile medical treatment if patients or their proxies are prepared to pay for it?. S Afr Med J.

[21] Vasques TCS, Lunardi VL, Silva PA, Carvalho KK, Lunardi Filho WD, Barros EJL (2016). Perception of nursing professionals about patient care of the terminally ill in the hospital environment. Texto Contexto Enferm.

[22] Kisorio LC, Langley GC (2016). Intensive care nurses' experiences of end-of-life care. Intensive Crit Care Nurs.

[23] Usberg G, Uibu E, Urban R, Kangasniemi M (2021). Ethical conflicts in nursing: an interview study. Nurs Ethics.

[24] Coombs M, Addington-Hall J, Long-Sutehall T (2012). Challenges in transition from intervention to end of life care in intensive care: a qualitative study. Int J Nurs Stud.

[25] Henrich NJ, Dodek PM, Alden L, Keenan SP, Reynolds S, Rodney P (2016). Causes of moral distress in the intensive care unit: a qualitative study. J Crit Care.

[26] Asayesh H, Mosavi M, Abdi M, Masoud MP, Jodaki K (2018). The relationship between futile care perception and moral distress among intensive care unit nurses. J Med Ethics Hist Med.

[27] Heydari A, Ahrari S, Chaharsoughi NT (2018). Moral distress in nursing and its contributors in the context of Iran. Health Spiritual Med Ethics.

[28] DeKeyser GF, Berkovitz K (2012). Surgical nurses' perceptions of ethical dilemmas, moral distress and quality of care. J Adv Nurs.

[29] Allie Z, Le Roux E, Mahlatsi K, Mofokeng B, Ramoo ZA, Sibiya K, Joubert G, Van Rooyen JP, Brits H (2018). Bereavement overload and its effects on, and related coping mechanisms of health care providers and ward administrators at National District Hospital in Bloemfontein, Free State. Afr J Prim Health Care Fam Med.

[30] Varcoe C, Pauly B, Storch J, Newton L, Makaroff K (2012). Nurses' perceptions of and responses to morally distressing situations. Nurs Ethics.

[31] Rafii F, Nikbakht Nasrabadi A, Karim MA (2016). End-of-life care provision: experiences of intensive care nurses in Iraq. Nurs Crit Care.

[32] Palmryd L, Rejnö Å, Godskesen TE (2021). Integrity at end of life in the intensive care unit: a qualitative study of nurses' views. Ann Intensive Care.

[33] Šaňáková Š, Čáp J (2019). Dignity from the nurses' and older patients' perspective: a qualitative literature review. Nurs Ethics.

[34] Efstathiou N, Ives J (2018). Compassionate care during withdrawal of treatment: a secondary analysis of ICU nurses' experiences. Nurs Ethics.

[35] Kongsuwan W, Locsin RC (2009). Promoting peaceful death in the intensive care unit in Thailand. Int Nurs Rev.

[36] Salter EK (2020). The new futility? The rhetoric and role of "suffering" in pediatric decision-making. Nurs Ethics.

[37] Fridh I, Forsberg A, Bergbom I (2009). Close relatives' experiences of caring and of the physical environment when a loved one dies in an ICU. Intensive Crit Care Nurs.

[38] Lamarche L, Bailey KA, Awan A, Risdon C, Pauw G, Vinoski TE (2020). Exploring primary care providers' understandings of body image in patient care. Body Image.

[39] Rainer J, Schneider JK, Lorenz RA (2018). Ethical dilemmas in nursing: an integrative review. J Clin Nurs.

